# Dataset on dynamics of Coronavirus on Twitter

**DOI:** 10.1016/j.dib.2020.105684

**Published:** 2020-05-08

**Authors:** Norman Aguilar-Gallegos, Leticia Elizabeth Romero-García, Enrique Genaro Martínez-González, Edgar Iván García-Sánchez, Jorge Aguilar-Ávila

**Affiliations:** a Centro de Investigaciones Económicas, Sociales y Tecnológicas de la Agroindustria y la Agricultura Mundial (CIESTAAM), Universidad Autónoma Chapingo (UACh), Chapingo, Estado de México, México; b Universidad Autónoma del Estado de México (UAEM), Estado de México, México; c Centro de Investigaciones Interdisciplinarias sobre Desarrollo Regional (CIISDER), Universidad Autónoma de Tlaxcala (UATx), Tlaxcala, México

**Keywords:** COVID-19, Pandemic, Infodemiology, Social media, Twitter, Retweets, Social Network Analysis, Hashtags

## Abstract

In this data article, we provide a dataset of 8,982,694 Twitter posts around the coronavirus health global crisis. The data were collected through the Twitter REST API search. We used the rtweet R package to download raw data. The term searched was “Coronavirus” which included the word itself and its hashtag version. We collected the data over 23 days, from January 21 to February 12, 2020. The dataset is multilingual, prevailing English, Spanish, and Portuguese. We include a new variable created from other four variables; it is called “type” of tweets, which is useful for showing the diversity of tweets and the dynamics of users on Twitter. The dataset comprises seven databases which can be analysed separately. On the other hand, they can be crossed to set other researches, among them, trends and relevance of different topics, types of tweets, the embeddedness of users and their profiles, the retweets dynamics, hashtag analysis, as well as to perform social network analysis. This dataset can attract the attention of researchers related to different fields on knowledge, such as data science, social science, network science, health informatics, tourism, infodemiology, and others.

Specifications table

**Table:** 

Subject	Social science, Health Informatics
Specific subject area	Social media
Type of data	Databases
How data were acquired	Twitter API search
Data format	Analysed, Filtered
Parameters for data collection	We searched for the term “Coronavirus” on Twitter posts.
Description of data collection	The raw data of the Twitter posts were downloaded from Twitter API search using the “rtweet (version 0.7.0)” R package. The collection process comprised from January 21 to February 12, 2020, i.e. 23 days. We searched for the term “Coronavirus”.
Data source location	CIESTAAM – UACh, Chapingo, Texcoco, Mexico.
Data accessibility	Mendeley Data: http://dx.doi.org/10.17632/7ph4nx8hnc.1

## Value of the data

• Different global diseases have attracted the attention in social media before. When an event of this magnitude happens, a massive quantity of people goes to these platforms to inform, exchange, post and look for information. This way, this dataset provides a great perspective on the dynamics of coronavirus on Twitter.
• Researchers on different fields of knowledge can use these databases to analyse the Twitter activity in the first stages of the coronavirus outbreak. Also, analysts or data scientists might be interested in the dataset to inform governments and agencies. This way, it would be possible to design effective strategies of communication and insertion on social media to face other potential pandemics.
• The dataset contains 8,982,694 Twitter posts around the coronavirus global crisis. Thus, it can be used to analyse different posting patterns and users’ dynamics, as well as diverse social networks, among other topics.
• The data collection started when there was a boom in the use of hashtags related to the coronavirus outbreak in China. Those hashtags became trending topics very quickly. This way, the dataset covers the initial dynamics of the coronavirus outbreak on Twitter.
• Seven filtered and analysed databases are provided, as well as a glance at their composition. Further analysis can be done by crossing these databases.
• A new variable, called “Type” of tweets, was created. This variable categorises each post into (1) tweets without mentions, (2) tweets with mentions, (3) retweets, and (4) replies. Through its use, it is possible to see different interactions and dynamics on Twitter.

## 1 Data Description

We conglomerate a dataset of 8,982,694 Twitter posts (tweets). These tweets reflect the early discussion around the coronavirus health global crisis on this social network. All the collected data were searched using the keyword “Coronavirus”. This implies that tweets containing this word were retrieved, as well as the posts including its hashtag version (#Coronavirus). The 8.98M were gathered from January 21 to February 12, 2020, i.e. 23 days. We selected those dates since, in the first one (21-Feb-2020), there was a boom around the topic on Twitter, where several hashtags related to the coronavirus and the outbreak in China were trending topics [1]. Then, the data collection was closed (12-February-2020) when the World Health Organization (WHO) changed the official name of this disease from “Coronavirus” to “COVID-19” (link to the official communication).

This paper compared to other publications, which also tracked diseases on Twitter, conglomerates a considerable quantity of Twitter posts within 23 days. For instance, Chew and Eysenbach [2] archived over 2 million tweets related to H1N1 or swine flu over eight months; while Stefanidis et al. [3] collected 6.25M tweets regarding the Zika outbreak for 12 weeks (three months). More recently, Cinelli et al. [4] analysed social media infodemic, and gathered around 1.18M posts on Twitter, in 18 days. Another example in another field of knowledge is the data on olympic-themed tweets, with 21.2M tweets collected over four months [5].

**Fig. 1:**
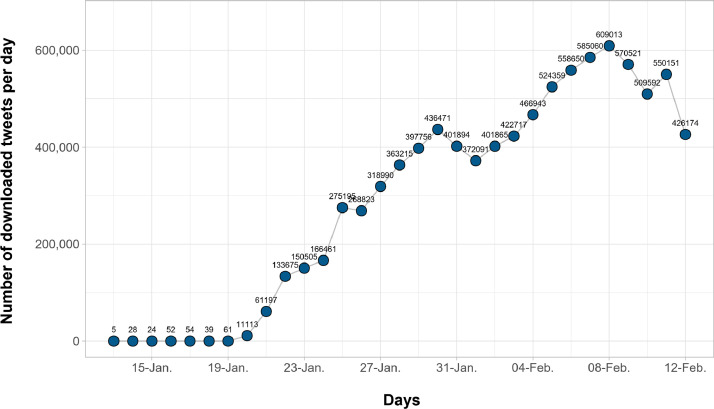
The trend of Twitter posts regarding the coronavirus topic. Note: Number of analysed tweets: 8,982,694; from 21-Jan to 12-Feb-2020.

Fig. 1 shows the daily distribution of the downloaded Twitter posts. It is possible to appreciate that the process of downloading data retrieved some tweets from 13-Jan-2020. It is worth mentioning that the amount of information that we could gather depended on two factors: (1) The increasing discussion of the topic on Twitter, and (2) The times in which the code for downloading the data was run. When we started to see the quantity of information on Twitter, the code was run as much as possible. This way, the trend in Fig. 1 shows that there was increasing attention on the topic on Twitter.

Since the raw data were vast, we had to filter and create different databases. All of them are available in a Mendeley dataset. This dataset has been created in line with Twitter's Terms & Conditions [6]; none of the databases contains the text of the collected tweets. Table 1 shows the name of each database and a brief description of its variables. It is worth mentioning that all the databases contain the variable “status_id”, which can be used to join them. This way, the Mendeley dataset can be further applied to cross databases and, thus, analyse different topics. It will depend on the objective of the analysis, or the researchers’ interests. If you have further questions about the dataset in Table 1 or you need more information about the raw variable, please contact us. Furthermore, through the “status_id” variable, it is possible to hydrated Twitter content using the Twitter APIs, and thus to get all the information related to the IDs.

**Table 1:** Databases in the Mendeley dataset. Note: When one variable was previously described, it is only mentioned in the next rows.

Database name	Description	Variables
01.Tw.Date.Lang	Date and time in which each Twitter post was created. Also, the language of each tweet. Approx size: 437 MB.	status_id: id of each Twitter post. created_at: date and time of posting. lang: languages of the tweets
02.Type.Tweets	Classification of each Twitter post regarding its nature. Four categories were used. Approx size: 274 MB.	status_id Type: each Twitter post was classified in 1. Tw: original tweets, without mentions; 2. MT: tweets with mentions; 3. RT: Retweets; 4. Re: Replies.
03.Links.Media. Tweets	To analyse two principal features of the Twitter posts: if they have URL and Media. For the analysis, the 3. RT posts were excluded. Approx size: 1.12 GB	status_id Type retweet_count: number of retweets received by each tweet. status_url: complete URL to the posts. urls_expanded_url: URL for external links in the posts. It was used to binarise when the post has a URL or not. media_expanded_url: URL for media in the posts. It was used to binarise when the post has a media (picture, gif, others) or not.
04.The.Most. Retweeted.0RT	The number of retweets received in each tweet. For the analysis, the databases were filtered, excluding the 3. RT tweets and those with 0 retweets. Thus, 955,364 tweets are included. Approx size: 99.3 MB	status_id screen_name: user/account who posted. Type retweet_count status_url
05.Users.And. Type	Twitter posts by user/account and its type. Approx size: 394 MB	status_id screen_name Type
06.Hashtags	Hashtags used in each post. For the analysis, the 3. RT posts were excluded. 32.8% of the rest posts include one or more hashtags. Approx size: 118 MB	status_id hashtags: words used as hashtags within each tweet.
07a.Tw.edges; 07b.Tw.nodes	List of edges and nodes for social network analysis. The list of edges includes the type of the post. Approx size: 427 and 51 MB, respectively.	For the Edges: from: source user/account of the link. to: target user/account of the link. Type status_id width: 1 by default For the Nodes: name: label of the user/account.

In the database “02.Type.Tweets”, a new variable was created based on other four variables which are not included in the dataset; but in the downloaded data they were: “is_retweet”, “reply_to_status_id”, “mentions_screen_name”, and “reply_to_user_id”. Since a user can tweet different messages with different characteristics, each one of the posts was classified in one of four categories, based on the following: 1. Tw, this was used for original tweets, when the post did not include any mention; 2. MT, this was created when the user mentioned other users within the tweet; 3. RT: Retweets, this category was used for those tweets which retweet one post and; 4. Re: Replies, this type of tweets were created when one user replied to another one.

**Fig. 2:**
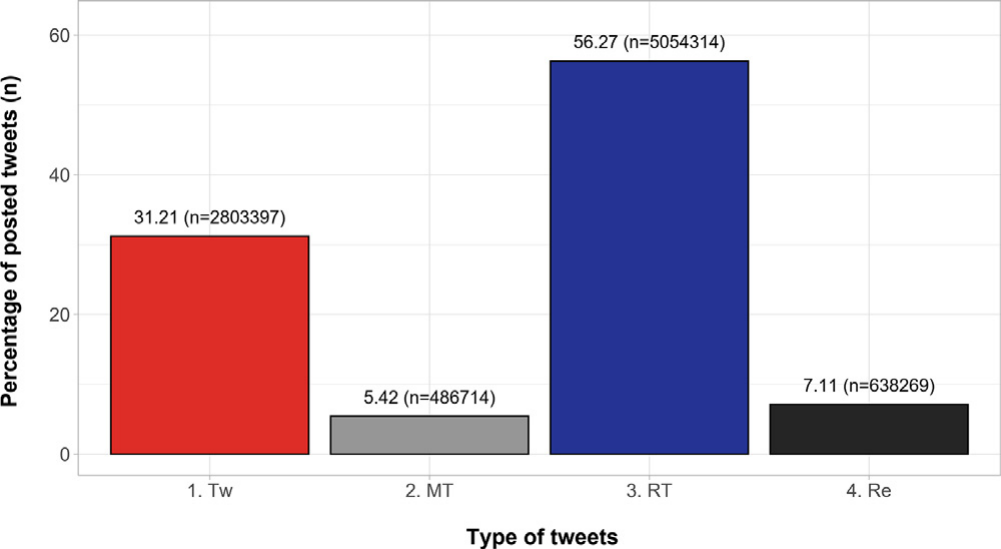
Type of Twitter posts regarding the coronavirus topic. Percentage and number (n) of tweets. Notes: Number of analysed tweets: 8,982,694; from 21-Jan to 12-Feb-2020. The types of tweets are 1. Tw. Original tweets (without mentions), 2. MT: Mentions, 3. RT: Retweets, and 4. Re: Replies.

By doing the above, in Fig. 2 it is possible to see that a lot of the interaction and discussion on Twitter around the coronavirus topic had been done by the retweets (category 3. RT). 56.3 % of the 8.98M Twitter posts were retweets. Fig. 2 also shows that users had been publishing original tweets without any mention within them (1. Tw, without mentions, 31.2%). The other databases and analyses provided in this paper are based on the differentiation of tweets by their “Type”. By including this variable in different databases (see Table 1), we want to prove that these categories influence a lot the interaction dynamics on Twitter. This, at the same time, enriches the applications and uses that the databases can have beyond this paper.

## 2 Experimental Design, Materials, and Methods

### 2.1 Data collection

The raw data of the 8.98M Twitter posts were downloaded from Twitter REST API search using the “rtweet (version 0.7.0)” R package, which was designed to simplify the interaction with Twitter's APIs and make it accessible to a wider range of users [7]. The collection process comprised from January 21 to February 12, 2020, i.e. 23 days. We searched for the term “Coronavirus” which included the word itself and its hashtag version (#Coronavirus). It is worth mentioning that the procedure to search tweets only returns data from the past 6-9 days, and it typically can return up to 18,000 Twitter statuses in a single call (see this link). Based on this, we run the process several times, creating a total of 424 files, which summed 17,974,805 records. We eliminated the duplicated ones, and the final number of retained tweets was 8,982,694, which are unique. All the collected data is multilingual since we did not use any kind of restriction about it. The most used languages on the tweets were English (en=56.8%), Spanish (es=19.4%), Portuguese (pt=5.1%), French (fr=4.7%) and, Italian (it=2.19); but in total, the tweets were written up in 65 different languages (Table 2).

**Table 2:** The most used languages in the Twitter posts.

id language	Language	Tweets	Accumulative tweets	Proportion (%)	Accumulative (%)
en	English	5,106,551	5,106,551	56.85	56.85
es	Spanish	1,739,335	6,845,886	19.36	76.21
pt	Portuguese	455,289	7,301,175	5.07	81.28
fr	French	420,665	7,721,840	4.68	85.96
it	Italian	196,670	7,918,510	2.19	88.15
und	Undefined	178,535	8,097,045	1.99	90.14
in	Indonesian	154,822	8,251,867	1.72	91.86
th	Thai	128,167	8,380,034	1.43	93.29
ja	Japanese	103,615	8,483,649	1.15	94.44
de	German	90,029	8,573,678	1.00	95.44
others (56)	Others	409,016	8,982,694	4.56	100.00

Since the raw data contained 90 variables, we had to filter and create different databases regarding the purpose of each analysis (see Table 1). This also enabled us to handle the data easier. We did this by taking into account Twitter's Terms & Conditions [6]. This way, in the next subsection, the results of analyses are shown related to each database explained above.

### 2.2 Links and media

**Fig. 3:**
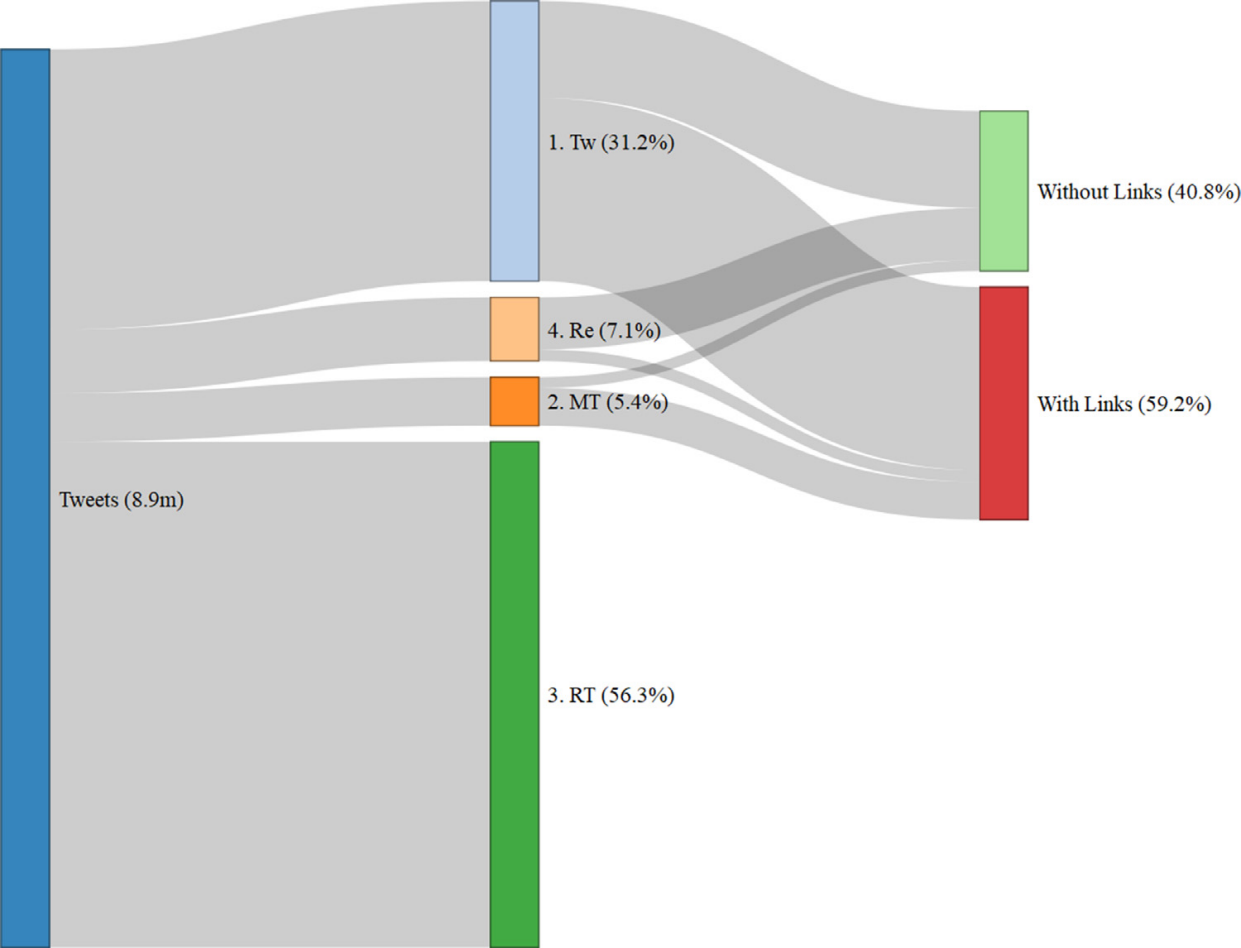
Twitter posts with links. Note: 1. Tw. Original tweets (without mentions), 2. MT: Mentions, 3. RT: Retweets, 4. Re: Replies. Categories 1. Tw, 2. MT, and 4. Re sum up 3,928,380 Twitter posts.

**Fig. 4:**
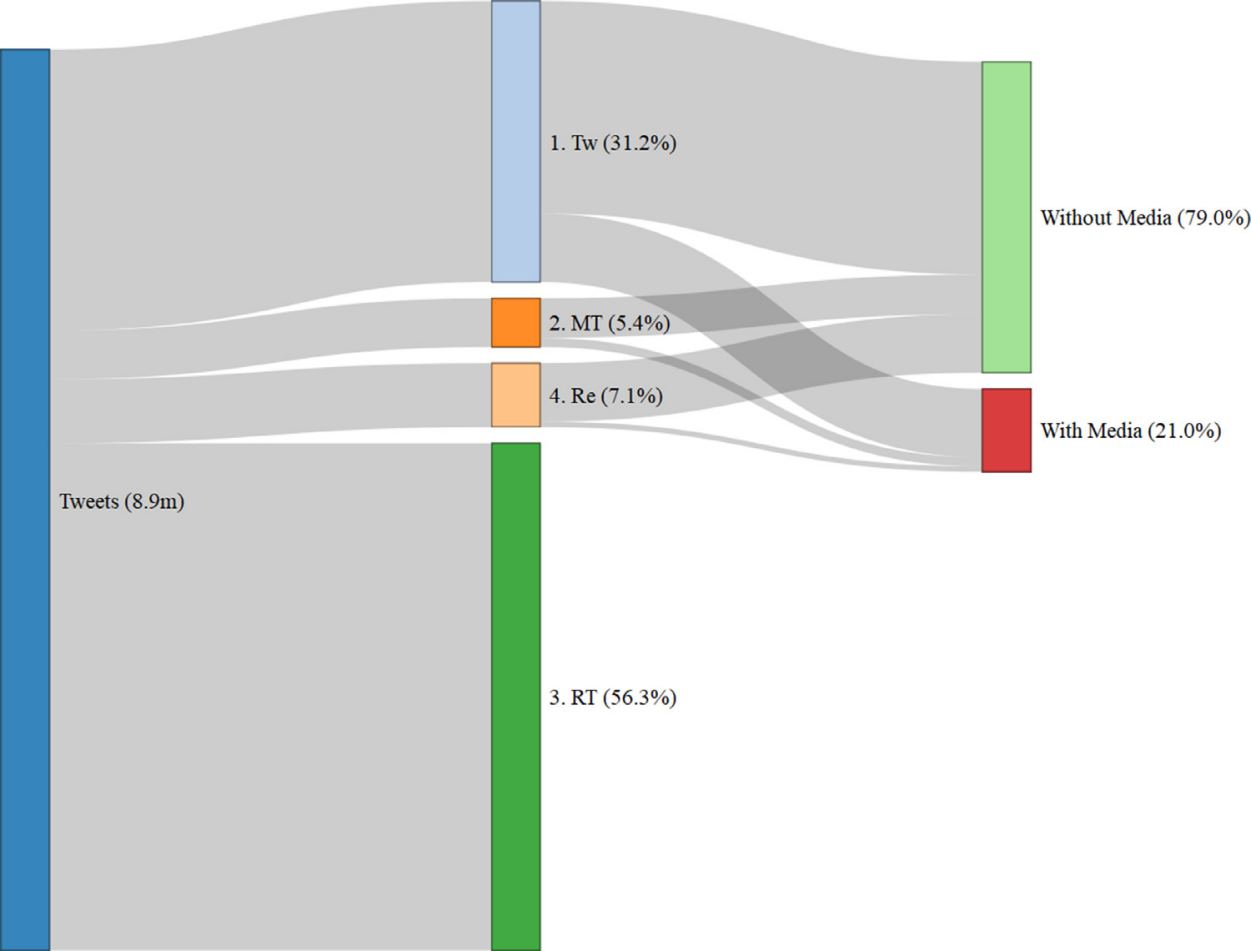
Twitter posts with media. Note: 1. Tw: Original tweets (without mentions), 2. MT: Mentions, 3. RT: Retweets, 4. Re: Replies. Categories 1. Tw, 2. MT, and 4. Re sum up 3,928,380 Twitter posts.

Two of the main features of Twitter posts are if they have links and media into the text. This is because tweets with these characteristics have more probability of being retweeted [8]. In order to explore that, we created a database called “03.Links.Media.Tweets” (see Table 1). This way, first, we excluded the posts whose type was “3. RT” (see Fig. 2); thus, 3,928,380 tweets (43.73%) were retained. We found that almost 60% of the Twitter posts contained a link; the tweets that quote other tweets are also included. Links were more used in the types 1. Tw, and 2. MT (Fig. 3). In the case of media, we conversely found that a considerable proportion (79%) of the tweets did not contain any kind of media (pictures, gifs, video, etc.). Twitter posts with media were more used in type 1. Tw (Fig. 4). Further information can be found in this database, which includes: the number of retweets received in each tweet; in this case, 75.7% of the 3,928,380 tweets did not receive any retweet. Also, it includes the complete URL to each post, the URL for the external links, as well as the URL for media.

### 2.3 The 25 tweets most retweeted

**Fig. 5:**
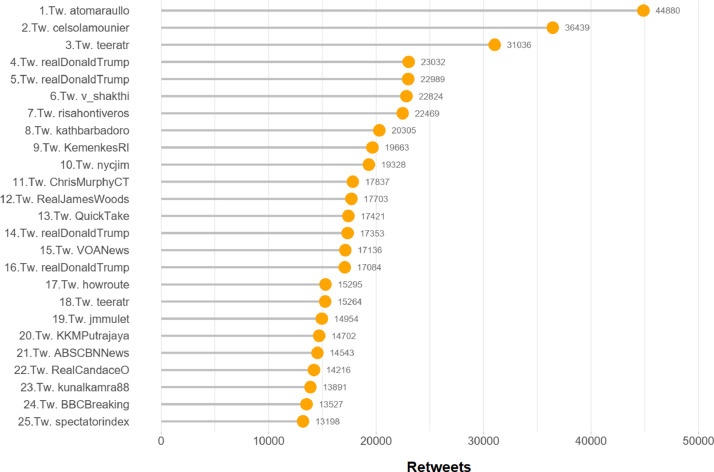
The 25 tweets most retweeted. Note: Y-axis refers to the source accounts of the tweet; prefix meaning Tw: Original tweets (1. Tw), MT: Mentions (2. MT), and Re: Replies (4. Re). To go to the tweets, follow the links, respectively: 1. @atomaraullo, 2. @celsolamounier, 3. @teeratr, 4. @realDonaldTrump, 5. @realDonaldTrump, 6. @v_shakthi, 7. @risahontiveros, 8. @kathbarbadoro, 9. @KemenkesRI, 10. @nycjim, 11. @ChrisMurphyCT, 12. @RealJamesWoods, 13. @QuickTake, 14. @realDonaldTrump, 15. @VOANews, 16. @realDonaldTrump, 17. @howroute, 18. @teeratr, 19. @jmmulet, 20. @KKMPutrajaya, 21. @ABSCBNNews, 22. @RealCandaceO, 23. @kunalkamra88, 24. @BBCBreaking, 25. @spectatorindex

Based on the database called “04.The.Most.Retweeted.0RT” (see Table 1), we analyse the number of retweets received in each tweet and, we delve deeply into the 25 tweets most retweeted. Fig. 5 shows that the most retweeted post had 44,800 retweets, and 13,198 other users retweeted the 25th post. We manually checked each tweet, as well as the accounts. By doing so, it was possible to determine that these tweets come from different actors, among them: Journalists (e.g., @atomaraullo), TV News Anchors (e.g., @teeratr), Politicians (e.g., @realDonaldTrump, @risahontiveros, @ChrisMurphyCT), Actors (e.g., @RealJamesWoods), News (e.g., @QuickTake, @VOANews), Official institutes (e.g., @KKMPutrajaya), Political activists (e.g., @ RealCandaceO) and, Stand-up comedians (e.g., @kunalkamra88).

It was interesting to see that these 25 tweets were on the type “1. Tw”, there was not any tweet type "2. MT" or "4. Re". Within these 25 tweets, the types of accounts are from very outstanding people or institutions; for instance, four tweets from @realDonaldTrump appear in Fig. 5. Another interesting fact is that the tweets in Fig. 5 were written in different languages, which highlight the relevance of this multilingual dataset. Other researchers can be interested in this information to explore the characteristics of the tweets and the number of retweets received.

### 2.4 Users and types of tweets

**Fig. 6:**
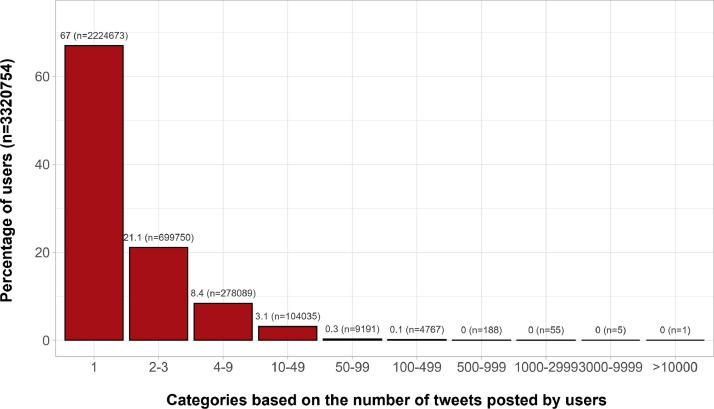
Percentage of users by the number of posted tweets. Note: Number of analysed tweets: 8,982,694, which were posted by 3,320,754 unique Twitter users.

In order to know how many users had been involved so far in the Twitter discussion around the coronavirus topic, we created a database called “05.Users.And.Type” (see Table 1). After analysing it, we found that 3,320,754 unique users had tweeted the 8.98M tweets. Interestingly, Fig. 6 shows that a considerable proportion of users tweeted only once (67%). But also, surprisingly, there was only one user that published more than 10,000 tweets.

**Fig. 7:**
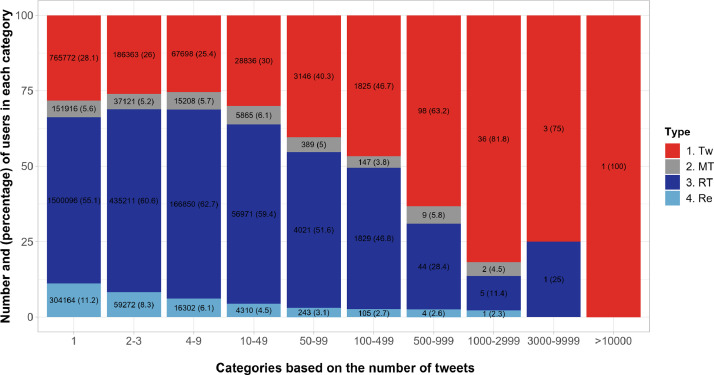
Number and percentage of users by categories and type of Twitter posts. Notes: 1. Tw: Original tweets (without mentions), 2. MT: Mentions, 3. RT, Retweets, and 4. Re: Replies. Number of analysed tweets: 8,982,694, which were tweeted by 3,320,754 unique users. The number of unique users differs from the sum of all the users in this plot (3,813,864) since one user can be classified into two or more categories due to the type and number of tweets.

Going more in-deep, when we included the types of tweets (Fig. 7), the analysis got enriched, and it was possible to see that more than 50% of the users who tweeted only once, they did it through the retweet (3. RT). 28% of the accounts that also posted once did it by using the tweet without mentions (1. Tw). Meanwhile, 11% replied to another tweet, and they did it in only one occasion. The last proportion was the type “2. MT”, with 5.6%. For the other categories based on the number of Twitter posts, the interpretation goes in the same direction. All this means that people on Twitter had been interacting by using different types of interaction (Tw, MT, RT, and Re); people were posting, others replying, and many others retweeting. These types of patterns can attract the attention of other researchers.

**Fig. 8:**
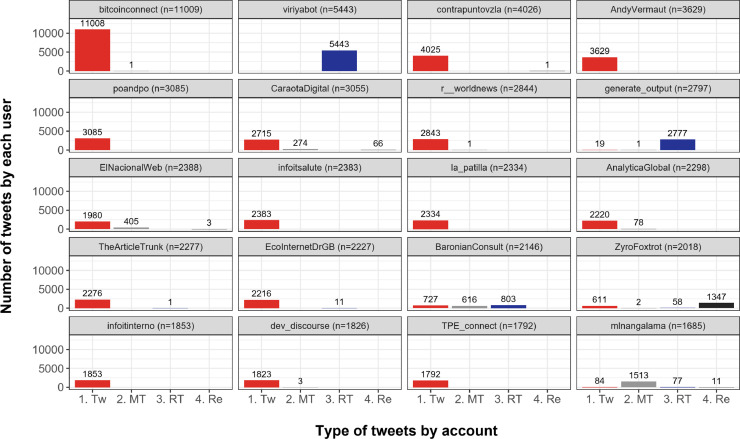
The 20 most active accounts by a total of tweets posted and type of tweets. Notes: 1. Tw: Original tweets (without mentions), 2. MT: Mentions, 3. RT: Retweets, and 4. Re: Replies. Number of analysed tweets: 8,982,694, which were posted by 3,320,754 unique Twitter users.

Following Fig. 6, it is possible to identify that there were 61 users more dynamic than the rest; they tweeted more than 1,000 tweets. But, when the variable of the types of tweets was introduced, in Fig. 7 we could appreciate that there were only 49 users. It shows that people who tweeted a lot was because they were mixing the types of interactions. Thus, they were classified into different categories in Fig. 7. To exemplify this type of mixing interaction, in Fig. 8 we present the 20 most active accounts by its total number of posts and the types of tweets. It is worth noting that neither account in Fig. 8 appeared in Fig. 5 and vice versa. This again highlights the different patterns of interactions on Twitter around the coronavirus topic.

### 2.5 Hashtags

The use of hashtags on Twitter posts is one of the primary mechanisms to get inserted into different trends and topics [9,10]. Thus, we created a database called “06.Hashtags” (see Table 1). First, we excluded the “3. RT” tweets type; then, we worked with 3,928,380 tweets. In this set of data, 1,289,328 (32.8%) tweets contained one or more hashtags into the text; the rest of the tweets (2,639,052 - 67.2%) did not include any kind of hashtag. Within these 1.28M tweets, we found 205,890 hashtags terms. But, 61.8% of those terms (127,270) were used only once. This way, we filtered the hashtags that were used more than 100 times; 2,189 terms were retained. Finally, we screened the hashtags that we considered as rare because they were based on numbers, symbols and were too long; 2,101 terms were obtained.

**Fig. 9:**
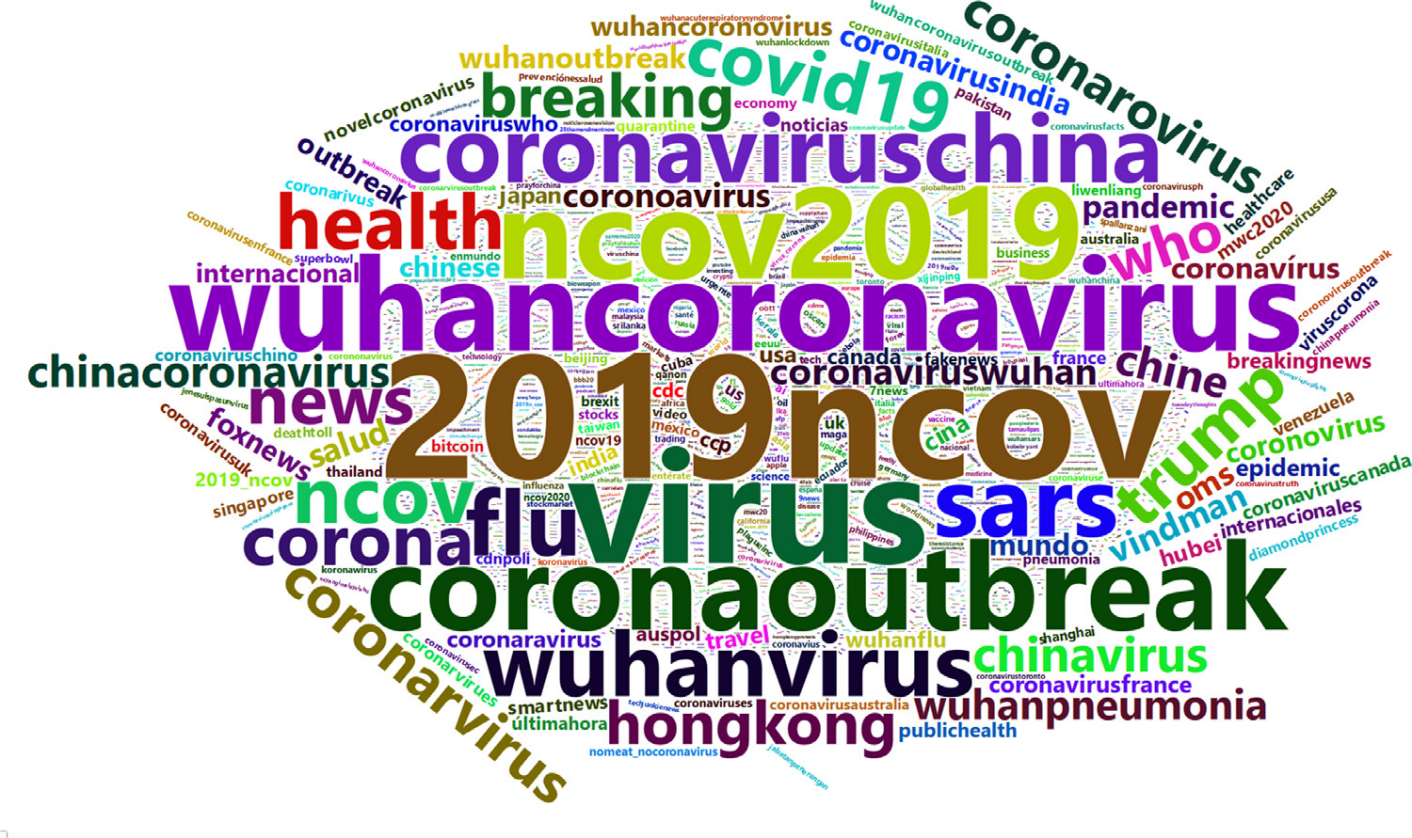
Wordcloud of hashtags used on the tweets around coronavirus topic.

By using a wordcloud, we show the diversity of multilingual hashtags used around the coronavirus topic (Fig. 9). Only for visual reasons, we removed four hashtags that were used more than 50,000 times because they distorted the wordcloud. They were: coronavirus, china, wuhan and, coronavirusoutbreak. In Table 3, the 50 hashtags most used are presented. We are sure that some researchers can be interested in the use of hashtags on the tweets; for instance, this can be applied in the infodemiology research topic (see Eysenbach [11]). Also, concerning the terms used on tweets (co-mentions), and how this affects the trends on Twitter, as well as the embeddedness of users.

**Table 3:** The 50 hashtags most used around the coronavirus topic on Twitter.

Hashtag	Freq.	Hashtag	Freq.	Hashtag	Freq.
1. coronavirus	959840	18. trump	11657	35. coronavirusindia	5042
2. china	121894	19. news	11498	36. coronavírus	4963
3. wuhan	73104	20. breaking	10055	37. wuhanoutbreak	4958
4. coronavirusoutbreak	50627	21. hongkong	9923	38. mundo	4899
5. 2019ncov	28538	22. coronarvirus	9602	39. foxnews	4864
6. virus	25836	23. who	9447	40. coronovirus	4811
7. wuhancoronavirus	21453	24. coronarovirus	8544	41. outbreak	4811
8. ncov2019	21086	25. chinavirus	8008	42. chinese	4783
9. coronaoutbreak	20123	26. chine	7468	43. wuhancoronovirus	4556
10. sars	16538	27. chinacoronavirus	7423	44. internacional	4396
11. coronaviruschina	15593	28. wuhanpneumonia	6929	45. cina	4094
12. flu	14915	29. coronaviruswuhan	6186	46. japan	4027
13. wuhanvirus	14763	30. vindman	5834	47. usa	4025
14. ncov	13332	31. salud	5590	48. coronaravirus	3988
15. health	12664	32. coronoavirus	5516	49. travel	3960
16. covid19	11917	33. pandemic	5411	50. coronavirusfrance	3905
17. corona	11775	34. oms	5397		

### 2.6 Social network analysis

A major contribution of this paper is that, from the 8.98M collected tweets, we constructed two databases for social network analysis (SNA); they were called “07a.Tw.edges” and “07b.Tw.nodes” (see Table 1). Both together will let other researchers study the network structures that emerge from the interaction among the Twitter users involved in the discussion of the coronavirus topic. SNA has been applied in other Twitter data-based papers, for instance [3,12,13]. SNA let us tackle and understand complex networks shaped by different actors, as well as visualise their links, and measure their characteristics [14,15].

Based on the two databases mentioned above, we got a network shaped by 3,565,497 nodes (Twitter users) and 7,296,841 links (without differentiating between mentions, retweets, and replies; but, in the “07a.Tw.edges” database this variable was included for further analysis). In this case, we used the “igraph (version 1.2.4.1)” R package [16]. Then, we simplified the links summing their recurrence and removing the loops. 3,565,497 nodes and 5,909,681 links formed the resulting network. The links are weighted since pairs of nodes could be linked in several times through different tweets. Also, we computed the number of components in the global network, and we found that 750,108 subnetworks constituted it. But, there were 645,305 components (86.0%) of size one; it means, those nodes did get any kind of interaction. The biggest component comprised 2,668,807 nodes (74.9% of the total) and 5,757,797 links (97.4% of the total). A diversity of different researchers could be interested in these two databases since they can analyse different types of interactions, networks structures, and node properties.

**Fig. 10:**
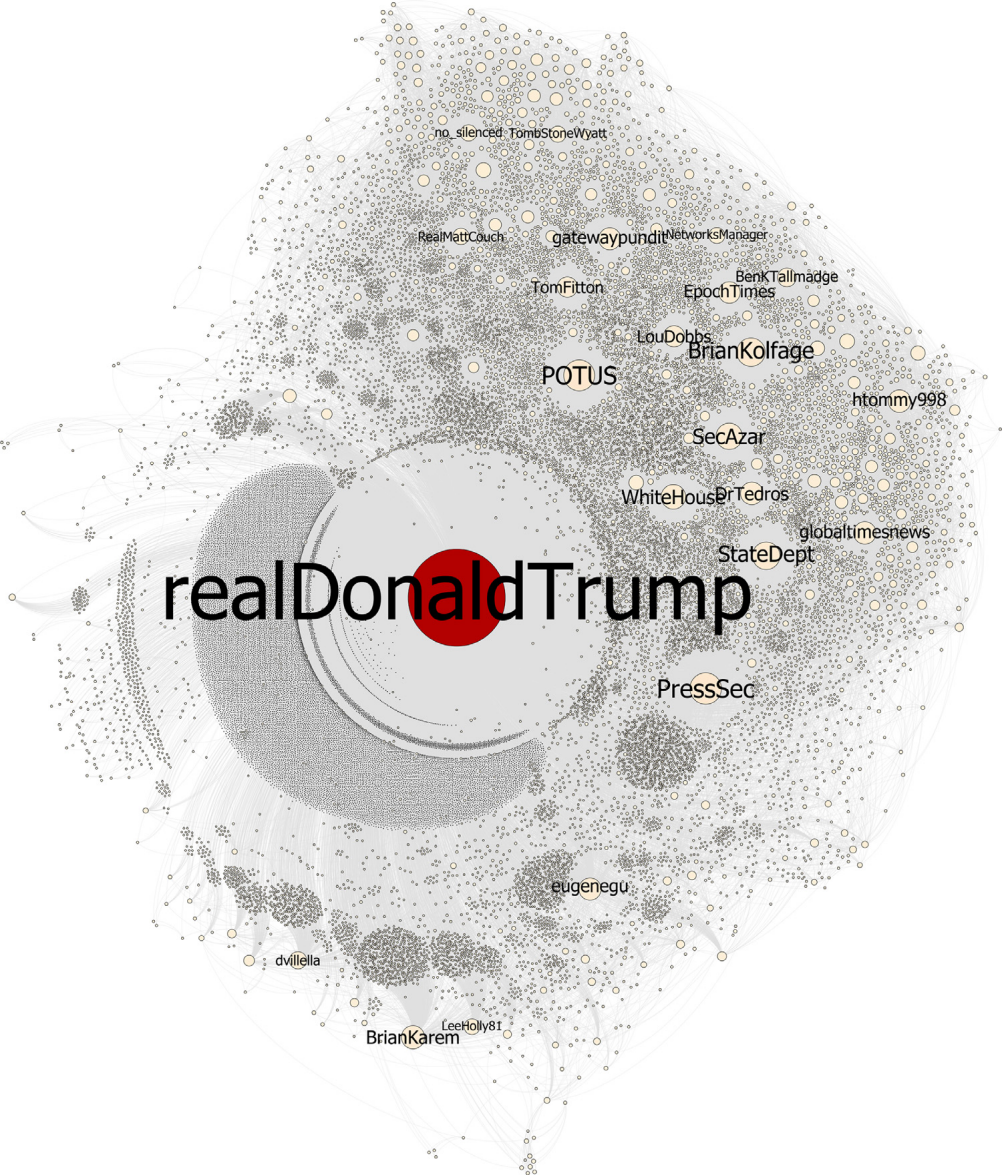
Social network around the account “@realDonaldTrump” on Twitter and related to the coronavirus topic.

In order to provide an example of the relevance of these databases and the use of an SNA approach, we analysed the network of the account: @realDonaldTrump. We filtered the database and got the users who interacted with this account, as well as how they interacted among them. Fig. 10 shows a network shaped by 30,197 nodes and 65,088 links. Interestingly, @realDonaldTrump tweeted only four tweets without any mention; but he was mentioned 3,719 times, retweeted 14,260 times and, replied 3,549 times. Fig. 10 also shows other prominent nodes based on the degree centrality.

## Declaration of Competing Interest

The authors declare that they have no known competing for financial interests or personal relationships which have, or could be perceived to have, influenced the work reported in this article.

## Appendix Supplementary materials

Image, application 1
